# The Quixotic Task of Forecasting Peaks of COVID-19: Rather Focus on Forward and Backward Projections

**DOI:** 10.3389/fpubh.2021.623521

**Published:** 2021-03-16

**Authors:** Ruy Freitas Reis, Rafael Sachetto Oliveira, Bárbara de Melo Quintela, Joventino de Oliveira Campos, Johnny Moreira Gomes, Bernardo Martins Rocha, Marcelo Lobosco, Rodrigo Weber dos Santos

**Affiliations:** ^1^Departamento de Ciência da Computação, Universidade Federal de Juiz de Fora, Juiz de Fora, Brazil; ^2^Departamento de Ciência da Computação, Universidade Federal de São João del-Rei, São João del-Rei, Brazil; ^3^Centro Federal de Educação Tecnológica de Minas de Gerais, Leopoldina, Brazil; ^4^Pós-Graduação em Modelagem Computacional, Universidade Federal de Juiz de Fora, Juiz de Fora, Brazil

**Keywords:** COVID-19, epidemiology, mathematical modeling, uncertainty quantification, projection, forecasting

## Abstract

Over the last months, mathematical models have been extensively used to help control the COVID-19 pandemic worldwide. Although extremely useful in many tasks, most models have performed poorly in forecasting the pandemic peaks. We investigate this common pitfall by forecasting four countries' pandemic peak: Austria, Germany, Italy, and South Korea. Far from the peaks, our models can forecast the pandemic dynamics 20 days ahead. Nevertheless, when calibrating our models close to the day of the pandemic peak, all forecasts fail. Uncertainty quantification and sensitivity analysis revealed the main obstacle: the misestimation of the transmission rate. Inverse uncertainty quantification has shown that significant changes in transmission rate commonly precede a peak. These changes are a key factor in forecasting the pandemic peak. Long forecasts of the pandemic peak are therefore undermined by the lack of models that can forecast changes in the transmission rate, i.e., how a particular society behaves, changes of mitigation policies, or how society chooses to respond to them. In addition, our studies revealed that even short forecasts of the pandemic peak are challenging. Backward projections have shown us that the correct estimation of any temporal change in the transmission rate is only possible many days ahead. Our results suggest that the distance between a change in the transmission rate and its correct identification in the curve of active infected cases can be as long as 15 days. This is intrinsic to the phenomenon and how it affects epidemic data: a new case is usually only reported after an incubation period followed by a delay associated with the test. In summary, our results suggest the phenomenon itself challenges the task of forecasting the peak of the COVID-19 pandemic when only epidemic data is available. Nevertheless, we show that exciting results can be obtained when using the same models to project different scenarios of reduced transmission rates. Therefore, our results highlight that mathematical modeling can help control COVID-19 pandemic by backward projections that characterize the phenomena' essential features and forward projections when different scenarios and strategies can be tested and used for decision-making.

## 1. Introduction

Epidemiology is defined in the International Epidemiological Association's dictionary as “the study of the occurrence and distribution of health-related events, states, and processes in specified populations, including the study of the determinants influencing such processes, and the application of this knowledge to control relevant health problems” (1). One of its main objectives, as stated in the definition, is to provide data so governments can plan and execute actions to prevent and control diseases. The current COVID-19 pandemic has put epidemiology at the center of the debate as, to date, there are no antivirals with proven efficacy against the disease (2–6). The first vaccines have just become available, but it is unknown how long does the immunity last after vaccination. Due to the lack of pharmaceutical treatments, non-pharmaceutical interventions suggested by epidemiologists have been used by many countries to deal with the pandemic, more specifically to reduce transmission and the impact on healthcare systems (7–12).

Mathematical and computational tools can be used by epidemiological studies to describe and predict the dynamics of the spread of a disease over time and space (13, 14). In addition, these tools can be used to assess the impact of non-pharmaceutical interventions, such as isolation (15–18). Several models have been proposed to describe the spread of diseases (14). Over the last months, many more have been developed to represent the dynamics of populations and their interactions, as well as to forecast the dynamics of the COVID-19 pandemic. Most describe the spread of COVID-19 based on ODEs (Ordinary Differential Equations) (17, 19–28), but statistical (29, 30), chaotic (27), and stochastic/probabilistic models (16, 18, 31, 32) have also been used.

Determining the pandemic's peak is a piece of valuable information for planning the health resource needed to cope with the disease. In the case of COVID-19, it is also relevant for economic reasons since many countries adopted lockdowns to reduce the spread of the disease, impairing their gross domestic product and, consequently, their budget while increasing their health and social protection costs systems. Although most of the models found in the literature can have their parameters adjusted to COVID-19 data, i.e., to describe the behavior of its spread in different cities, regions, or countries, they usually fail to forecast the peak of the pandemic accurately (26–28). One could ask if one particular modeling technique would be more appropriate to forecast the dynamics of COVID-19 than others. However, a review of the literature does not clearly show that this hypothesis holds. In fact, in the literature we can find examples of forecasts that failed using SEIR (26), SIRD (25) extensions of SEIR with more compartments (28), statistical (33, 34), agent-based (35), machine-learning (36), and chaos-based theory models (27).

Another possible explanation for the failed forecasts could be related to the classical problem of overfitting (37), where the model can replicate the data it is adjusted to but fails on any attempt of extrapolation or forecasting. In this paper, we took several precautions to prevent the issue of overfitting. A simple mathematical model, based on the classical SIRD model, was adopted with a reduced number of parameters. We decided to keep the model as simple as possible since adding more compartments increases the number of unknown parameters to be estimated, which hinders the accurate calibration of the model. We also used the methods of forward and inverse uncertainty quantification (UQ). The parameters of the models were treated as probability density functions (PDFs) during the task of model-to-data fitting (via inverse UQ) and during the tasks of forecasting and projections (via forward UQ). In addition, during the fitting phase, we also considered a possible discrepancy between model and reality (38). Nevertheless, in this paper, we show that the above precautions to avoid overfitting did not solve the problem of mispredicting the peak of COVID-19.

This work shows that this common pitfall is likely due to fast and unpredictable changes in the disease's transmission rate. The models are useful for predictions in a more controlled environment. It is like trying to predict the trajectory of a paper aeroplan on a windless day. It is much easier than during a storm. Likewise, it is challenging to predict significant changes in how a particular society behaves, mitigation policies, or how society responds to them during a pandemic. However, these all have a direct impact on the transmission rate, which in turn significantly affects the dynamics of the pandemic, as shown in previous studies (20, 39, 40). Nevertheless, differently from the weather, mitigation policies can be planned and controlled to some extent. This fact brings up the importance of projections of different scenarios during this pandemic. Different from forecasts, projections aim to study one or more hypothetical scenarios. In contrast, forecasts use the available data and try to predict future trends (41).

The models and techniques used in this work were first described in previous work (20). The model consists of a non-linear system of ordinary differential equations subject to uncertainty in some of its parameters and initial conditions. Probability density functions (PDFs) were used to describe the uncertainties associated with these parameters, so they are not scalars. Some of them, such as the transmission rate, are additionally time-dependent. After we adjusted the model's PDFs to a particular data (via inverse Uncertainty Quantification), the model was able to provide useful insights in terms of characterization of the pandemic dynamics in a particular country.

The current study uses four countries as examples, with distinct population sizes and demographics: Austria, Germany, Italy, and South Korea. For each country, we first show that the proposed model and methods correctly described the dynamics of total reported cases, active infected, and deaths when fitting model to data, i.e., our models can reproduce the different dynamics and peaks. Next, we show that our models can forecast the pandemic dynamics 20–30 days ahead when far from the peaks. However, all pandemic peaks' forecasts fail, even when adjusting the model to the data up to 5 days before the peak in each country. We further investigate this weakness, which is shared by many distinct models presented in the literature, by analyzing which parameter was misestimated via backward projection or inverse UQ. The analysis pointed to the misestimation of changes in the transmission rate near the peak as the primary source of error. In addition, the correct estimation of any temporal change in the transmission rate was only possible many days ahead.

Finally, we performed projections, adjusted the model to the data up to 10 days before the peak, and focused on different scenarios that considered changes in the transmission rate. The projections that simulated significant reductions in the transmission rate were the ones where the pandemic peaks were closest to the real observed ones.

Therefore, our results highlight how mathematical models can help the fight against the COVID-19 pandemic: by characterizing important parameters that dictate the pandemic dynamics, as performed before in our previous work (20); and via projections, when different scenarios and strategies can be tested and used for decision-making. In addition, our analysis suggests that the misestimation of changes in the transmission rate near the peaks is the main source of error during the task of forecasting the peaks of COVID-19 pandemic.

## 2. Materials and Methods

To demonstrate the impact of the transmission rate in forecasting the peak of COVID-19 pandemic, the parameters of our model (20) were calibrated according to total and active COVID-19 cases and deaths in three countries that have already achieved the pandemic peak: Austria, Germany, South Korea, and Italy. Peak predictions are performed considering a distinct number of available days.

The characterization of COVID-19 in these four countries is performed using inverse UQ techniques. Therefore, during the calibration of the model, the coefficients are treated as unknown probability density functions. Once estimated, the PDFs of the coefficients, their means, standard deviations (SD), and shape provide important information on model parameters that are essential in the characterization of the COVID-19 pandemic. The model and how it is adjusted are briefly described in this section to facilitate the understanding of the results. More details about the model, calibration of the parameters, and uncertainties can be found in our previous work (20).

### 2.1. Mathematical Model

The model used in this work (20) is based on the classic compartmental SIRD model (13, 14, 42–44), and was kept as simple as possible to reduce the number of unknown parameters to be estimated.

The model is described by the following set of equations:

(1) $$\left\{ \begin{array}{l} \frac{dS}{dt}\;=-\frac{\alpha (t)}{N}SI,\\ \frac{dI}{dt}\;=\frac{\alpha (t)}{N}SI-\beta I-\gamma I,\\ \frac{dR}{dt}\;=\gamma I,\\ \frac{dD}{dt}\;=\beta I,\\ I_{r}\;=\theta I,\\ R_{r}\;=\theta R,\\ C\;=I_{r}+R_{r}+D, \end{array}\right.$$

where *S*, *I*, *R*, *D*, *I*_*r*_, *R*_*r*_, and *C* are the variables that represent the number of individuals within a population of size *N* that are susceptible, infected, recovered, dead, reported as infected, reported as recovered, and total confirmed cases, respectively. The term α(*t*) = *a*(*t*)*b* denotes the rate at which a susceptible individual becomes infected; where *a*(*t*) denotes the probability of contact and *b* the rate of infection. The function *a*(*t*) models temporal changes in the transmission rate:

(2) $$a(t)\;=\left\{ \begin{array}{l} 1,\text{ if }t<t_{i},\\ \frac{r-1}{\Delta }(t-t_{i})+1,\text{ if }t_{i}\leq t\leq t_{i}+\Delta ,\\ r,\text{ otherwise}. \end{array}\right.$$

Each transmission change starts at *t*_*i*_, and is changed by a factor *r* at the final time *t*_*i*_+Δ. The mortality rate of infected individuals is modeled by the constant *β* = *m*(1/*τ*_*o*_), where *m* is the probability of death. It must be noted that this is not the same as the rate of death and as the percentage of death among the reported cases of positive infection (*I*_*r*_). The number of days from infection until death is represented by *τ*_*o*_ = *τ*_1_ + *τ*_2_, where *τ*_1_ is the incubation time of the virus and *τ*_2_ is the time between the first symptoms until death. Similarly, represented by *τ*_*r*_ = *τ*_1_ + *τ*_3_, where *τ*_*r*_ is the number of days from infection until recovery and *τ*_3_ is the time between the first symptoms until recovery. The rate at which infected individuals recover from the virus is given by constant γ = (1−*m*)(1/*τ*_*r*_). Lastly, the percentage of confirmed infected individuals that are notified or reported is represented by θ.

For making projections using the model beyond the last day used during the fitting, we consider α(*t*) = *a*_*p*_(*t*)*b*:

(3) $$a_{p}\text{(t) =}\left\{ \begin{array}{l} 1,\text{ if }t<t_{i},\\ \frac{r-1}{\Delta }(t-t_{i})+1,\text{ if }t_{i}\leq t\leq t_{i}+\Delta ,\\ r,\text{ if }t_{i}+\Delta <t\leq t_{f},\\ \frac{r_{f}-r}{\Delta_{f}}(t-t_{f})+r,\text{ if }t_{f}<t\leq t_{f}+\Delta_{f},\\ r_{f},\text{ otherwise.} \end{array}\right.$$

**Figure 1:**
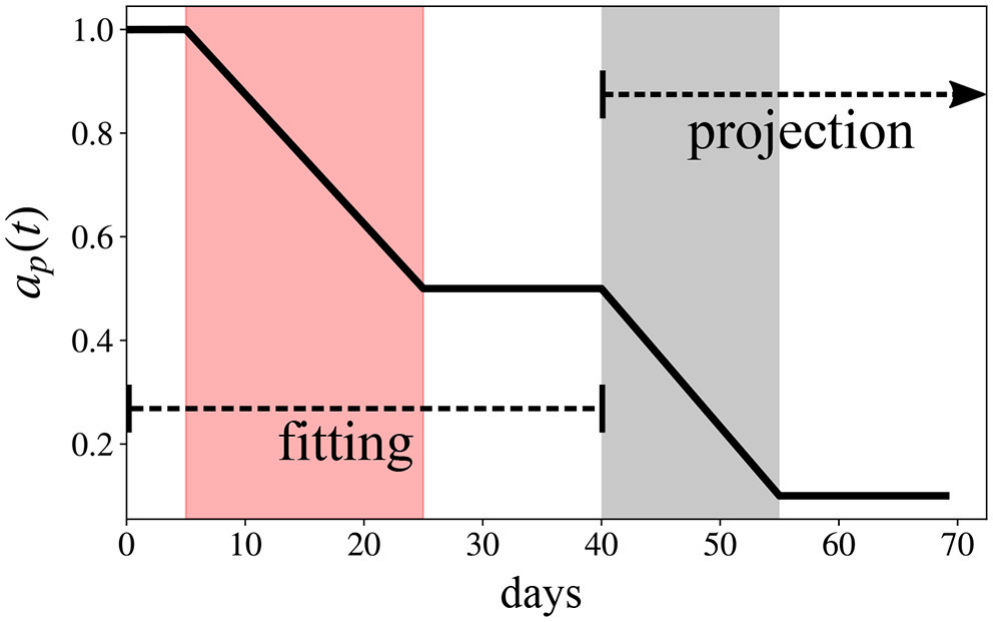
Illustrative example of Equation (3), *a*_*p*_(*t*), considering *t*_*i*_ = 5, Δ = 20 days, *t*_*f*_ = 20, *r* = 0.5, *r*_*f*_ = 0.1, and Δ_*f*_ = 15 days for a 70 days simulation.

The function *a*_*p*_(*t*) is similar to *a*(*t*) but it adds different scenarios in terms of how the transmission rate evolves after the last day of data used for model fitting. The constant *t*_*f*_ is the last day used during the calibration of the model, *r*_*f*_ is the final value of *a*_*p*_(*t*) during the projection phase, and Δ_*f*_ is the time interval for *a*_*p*_(*t*) to change from *r* to *r*_*f*_. Figure 1 is an illustrative example for Equation (3).

### 2.2. Parameter Estimation and Uncertainty Quantification

Model parameters were adjusted using the differential evolution (DE) optimization method (45) implemented in the C programming language. The DE was used to estimate each of the parameters of the proposed mathematical model, respecting the limits established for each one of them [see (20)]. The parameter values were estimated based on official data from the epidemic reported in each country (Austria, Germany, S. Korea, and Italy) and made available by Dong et al. (46). Here, we are using *Î*(*t*) as the reported numbers of active cases, $\hat{D}\left(t\right)$ the number of deaths, and *Ĉ*(*t*) the total confirmed cases. The following objective function, Equation (5), was used to minimize the relative error ($R_{E}\left(\lambda ,\hat{\lambda }\right)$) between the data and the model:

(4) $$\begin{array}{r} R_{E}\left(\lambda ,\hat{\lambda }\right)=\frac{∥\lambda \left(t,\;p\right)-\hat{\lambda }\left(t\right){∥}_{1}}{∥\hat{\lambda }\left(t\right){∥}_{1}}, \end{array}$$

(5) $$\begin{array}{r} \underset{p}{\min}\left(\omega_{1}R_{E}\left(I,\hat{I}\right)+\omega_{2}R_{E}\left(D,\hat{D}\right)+\omega_{3}R_{E}\left(C,\hat{C}\right)\right), \end{array}$$

where *p* is the set of parameters to be estimated and *ω*_*n*_ is a weight. For this work, we used *ω*_1_ = *ω*_2_ = *ω*_3_ = 1.0.

Some input parameters of the model are subject to uncertainties and variations, due to measurement errors, technical limitations, and resource availability. Parameters such as the incubation period, mortality, period from symptoms to death, period from symptoms to recovery, and the effectiveness of contact reduction are hampered by uncertainties, as suggested in data from literature. Therefore, when evaluating models such as the one studied in this work, it is important to evaluate the uncertainties from estimated input parameters, providing a confidence interval for the predictions.

Although the parameter intervals are reported in the literature, we still do not know how their probability densities functions behave. So, an inverse UQ technique was used to estimate the PDFs and corresponding uncertainties of the input parameters or coefficients of the model during model calibration. For each model parameter, we determined its PDF from the fitting procedure using the DE method. Among the offsprings generated by the DE, during the fitting process, we selected individuals with *o*(*p*) ≤ 25%, where *o*(*p*) is defined in Equation (6).

(6) $$o(p)=\underset{p}{\max}\left(\omega_{n}R_{E}(I,\hat{I}),\omega_{n}R_{E}(D,\hat{D}),\omega_{n}R_{E}(C,\hat{C}\right),$$

in which *p* is the set of parameters to be estimated and *ω*_*n*_ are the same weights defined in Equation (5). Using this procedure, we acknowledge a possible discrepancy of up to 25% between the model and reality.

Thus, from these samples, we estimate the covariance matrix and mean of all parameters. We use these data to generate a multivariate normal distribution to perform a forward UQ analysis via the Monte Carlo method with a total of 10,000 samples using the ChaosPy library (47).

Finally, we evaluate how the uncertainties in the input parameters of the model impact its outputs using forward UQ techniques. The forward UQ technique propagates the uncertainty of the input parameters to the outputs. Among the consolidated methods from the literature, Monte Carlo is one of the most used to perform uncertainty propagation (48–50). Briefly, this method draws samples of the input parameters and evaluates the model using them to provide statistical properties for the quantities of interest.

### 2.3. Data Sources

The model was calibrated using the data reported by the Center for Systems Science and Engineering at Johns Hopkins University (46), between 01/22/2020 and 12/20/2020. The bounds used for the parameters are described in (20).

### 2.4. Characterization

Tables 1–4 show the mean and standard deviation of all offspring solution with *o*(*p*) ≤ 25%. The value of *N* is 9.00 × 10^6^ for Austria, 83.02 × 10^6^ for Germany, 60.42 × 10^6^ for Italy and 51.47 × 10^6^ for South Korea.

**Table 1:** Values of parameters used to fit model to data.

**Symbol**	**Austria**		**Germany**		**S. Korea**		**Italy**	
	**Mean**	**STD**	**Mean**	**STD**	**Mean**	**STD**	**Mean**	**STD**
*b*	3.01 × 10^−1^	5.98 × 10^−3^	3.35 × 10^−1^	4.45 × 10^−3^	5.01 × 10^−1^	8.19 × 10^−3^	6.40 × 10^−2^	5.39 × 10^−4^
*r*	1.05 × 10^−2^	2.78 × 10^−3^	8.19 × 10^−2^	3.73 × 10^−3^	1.01 × 10^−2^	5.96 × 10^−4^	2.75 × 10^−1^	2.81 × 10^−2^
*t*_*i*_	1.04 × 10^1^	1.06 × 10^0^	9.16 × 10^0^	9.47 × 10^−1^	5.53 × 10^−1^	3.63 × 10^−1^	9.47 × 10^1^	2.21 × 10^0^
Δ	1.85 × 10^1^	1.47 × 10^0^	3.20 × 10^1^	1.23 × 10^0^	1.92 × 10^1^	4.66 × 10^−1^	5.48 × 10^0^	3.90 × 10^0^
θ	8.95 × 10^−1^	1.80 × 10^−1^	6.38 × 10^−1^	2.72 × 10^−2^	9.71 × 10^−1^	6.21 × 10^−2^	9.75 × 10^−1^	9.76 × 10^−2^
τ_1_	3.20 × 10^0^	9.81 × 10^−1^	9.33 × 10^0^	2.18 × 10^−1^	1.31 × 10^1^	3.77 × 10^−1^	1.60 × 10^1^	3.71 × 10^0^
τ_2_	1.17 × 10^1^	2.05 × 10^0^	6.26 × 10^0^	4.71 × 10^−1^	1.74 × 10^1^	2.04 × 10^0^	2.47 × 10^1^	4.27 × 10^0^
τ_3_	1.56 × 10^1^	9.20 × 10^−1^	7.15 × 10^0^	1.11 × 10^−1^	1.64 × 10^1^	3.47 × 10^−1^	2.85 × 10^1^	3.44 × 10^0^
*m*	3.09 × 10^−2^	4.45 × 10^−3^	3.13 × 10^−2^	9.88 × 10^−4^	2.75 × 10^−2^	2.49 × 10^−3^	3.01 × 10^−2^	2.85 × 10^−3^

*b, COVID-19 transmission rate; m, death probability; r, contact reduction; t_i_, start of intervention policy; Δ, duration of intervention policy; τ_1_, incubation period; τ_2_, period from symptoms to death; τ_3_, period from symptoms to recovery; θ, fraction of notified cases. The model parameters were calibrated using data from the first day with more than 100 cases to 05/11/2020 for each country for Austria, Germany, and S. Korea. For Italy the model parameters were calibrated from 08/17/2020 to 12/14/2020*.

**Table 2:** Value of parameters used to make short forecasts.

**Symbol**	**Austria**		**Germany**		**S. Korea**		**Italy**	
	**Mean**	**STD**	**Mean**	**STD**	**Mean**	**STD**	**Mean**	**STD**
*b*	7.59 × 10^−2^	3.57 × 10^−3^	5.02 × 10^−2^	2.73 × 10^−3^	6.27 × 10^−2^	1.10 × 10^−3^	1.90 × 10^−2^	1.59 × 10^−3^
*r*	1.96 × 10^0^	9.81 × 10^−2^	2.07 × 10^0^	1.15 × 10^−1^	1.83 × 10^0^	1.62 × 10^−1^	4.10 × 10^−1^	1.43 × 10^−1^
*t*_*i*_	7.51 × 10^−1^	2.97 × 10^0^	1.50 × 10^1^	1.14 × 10^0^	2.63 × 10^1^	1.45 × 10^0^	5.48 × 10^−1^	1.75 × 10^0^
Δ	2.85 × 10^1^	3.43 × 10^0^	1.26 × 10^1^	1.33 × 10^0^	1.83 × 10^0^	1.34 × 10^0^	1.19 × 10^1^	2.06 × 10^0^
θ	3.90 × 10^−1^	3.21 × 10^−2^	2.77 × 10^−1^	4.00 × 10^−2^	3.85 × 10^−1^	3.45 × 10^−2^	6.49 × 10^−1^	5.76 × 10^−2^
τ_1_	4.33 × 10^0^	1.23 × 10^0^	5.20 × 10^0^	1.70 × 10^0^	8.99 × 10^0^	2.61 × 10^0^	7.95 × 10^0^	3.05 × 10^0^
τ_2_	1.63 × 10^1^	3.00 × 10^0^	1.75 × 10^1^	2.21 × 10^0^	1.67 × 10^1^	2.86 × 10^0^	1.74 × 10^1^	3.92 × 10^0^
τ_3_	9.28 × 10^0^	1.22 × 10^0^	9.91 × 10^0^	1.63 × 10^0^	1.18 × 10^1^	2.52 × 10^0^	1.97 × 10^1^	3.61 × 10^0^
*m*	4.33 × 10^−3^	7.07 × 10^−4^	5.62 × 10^−3^	1.65 × 10^−3^	1.00 × 10^−2^	1.21 × 10^−3^	4.27 × 10^−2^	3.61 × 10^−3^

*b, COVID-19 transmission rate; m, death probability; r, contact reduction; t_i_, start of intervention policy; Δ, duration of intervention policy; τ_1_, incubation period; τ_2_, period from symptoms to death; τ_3_, period from symptoms to recovery; θ, fraction of notified cases. The model parameters were calibrated using 30 days of data. The model was then used to forecast the following 30 days for Austria, Germany, S. Korea, and Italy*.

**Table 3:** Value of parameters used to predict and to project the pandemic peak considering 5 days before the peak.

**Symbol**	**Austria**		**Germany**		**S. Korea**		**Italy**	
	**Mean**	**STD**	**Mean**	**STD**	**Mean**	**STD**	**Mean**	**STD**
*b*	2.94 × 10^−1^	2.10 × 10^−3^	3.02 × 10^−1^	1.36 × 10^−3^	5.88 × 10^−1^	5.69 × 10^−3^	4.61 × 10^−2^	1.97 × 10^−4^
*r*	3.95 × 10^−1^	1.74 × 10^−2^	2.10 × 10^−1^	9.83 × 10^−3^	1.58 × 10^−1^	6.25 × 10^−3^	1.77 × 10^0^	1.48 × 10^−2^
*t*_*i*_	9.07 × 10^0^	5.62 × 10^−1^	1.32 × 10^1^	4.24 × 10^−1^	5.53 × 10^−2^	1.96 × 10^−1^	4.87 × 10^1^	1.00 × 10^0^
Δ	1.19 × 10^1^	5.88 × 10^−1^	1.78 × 10^1^	4.44 × 10^−1^	1.34 × 10^1^	3.13 × 10^−1^	2.52 × 10^0^	1.70 × 10^0^
θ	8.52 × 10^−1^	3.42 × 10^−2^	6.81 × 10^−1^	2.35 × 10^−2^	9.25 × 10^−1^	4.22 × 10^−2^	1.52 × 10^−1^	2.37 × 10^−2^
τ_1_	1.40 × 10^1^	1.66 × 10^−1^	1.40 × 10^1^	2.16 × 10^−1^	1.40 × 10^1^	1.91 × 10^−1^	3.96 × 10^1^	1.36 × 10^0^
τ_2_	1.77 × 10^1^	1.33 × 10^0^	2.14 × 10^1^	1.10 × 10^0^	1.97 × 10^1^	1.18 × 10^0^	5.94 × 10^1^	3.02 × 10^0^
τ_3_	1.70 × 10^1^	1.92 × 10^−1^	1.69 × 10^1^	2.86 × 10^−1^	1.70 × 10^1^	1.22 × 10^−1^	2.09 × 10^1^	1.82 × 10^0^
*m*	3.39 × 10^−2^	6.41 × 10^−4^	3.38 × 10^−2^	5.69 × 10^−4^	3.35 × 10^−2^	1.87 × 10^−3^	1.01 × 10^−2^	7.36 × 10^−4^

*b, COVID-19 transmission rate; m, death probability; r, contact reduction; t_i_, start of intervention policy; Δ, duration of intervention policy; τ_1_, incubation period; τ_2_, period from symptoms to death; τ_3_, period from symptoms to recovery; θ, fraction of notified cases. The model parameters were calibrated using data from the first day with more than 100 cases to 5 days before the pandemic peak for Austria, Germany, and S. Korea. For Italy the model parameters were calibrated from 08/17/2020 to 5 days before the second peak*.

**Table 4:** Value of parameters used to predict and to project the pandemic peak considering 10 days before the peak.

**Symbol**	**Austria**		**Germany**		**S. Korea**		**Italy**	
	**Mean**	**STD**	**Mean**	**STD**	**Mean**	**STD**	**Mean**	**STD**
*b*	3.15 × 10^−1^	1.97 × 10^−3^	3.04 × 10^−1^	1.33 × 10^−3^	5.87 × 10^−1^	4.20 × 10^−3^	4.56 × 10^−2^	9.43 × 10^−5^
*r*	5.97 × 10^−1^	1.72 × 10^−2^	2.90 × 10^−1^	2.38 × 10^−2^	1.29 × 10^−1^	5.29 × 10^−3^	1.92 × 10^0^	7.42 × 10^−3^
*t*_*i*_	4.73 × 10^0^	5.58 × 10^−1^	1.46 × 10^1^	4.06 × 10^−1^	2.76 × 10^−2^	1.38 × 10^−1^	5.12 × 10^1^	4.55 × 10^−1^
Δ	1.13 × 10^1^	5.62 × 10^−1^	1.14 × 10^1^	4.50 × 10^−1^	1.40 × 10^1^	1.42 × 10^−1^	3.07 × 10^0^	8.16 × 10^−1^
θ	9.96 × 10^−1^	1.92 × 10^−2^	9.76 × 10^−1^	2.83 × 10^−2^	8.38 × 10^−1^	3.57 × 10^−2^	1.74 × 10^−1^	3.23 × 10^−2^
τ_1_	1.40 × 10^1^	3.00 × 10^−1^	1.40 × 10^1^	2.65 × 10^−1^	1.40 × 10^1^	1.91 × 10^−1^	3.93 × 10^1^	1.53 × 10^0^
τ_2_	2.18 × 10^1^	7.73 × 10^−1^	2.16 × 10^1^	9.60 × 10^−1^	1.67 × 10^1^	1.26 × 10^0^	5.90 × 10^1^	3.10 × 10^0^
τ_3_	1.70 × 10^1^	2.44 × 10^−1^	1.69 × 10^1^	3.97 × 10^−1^	1.70 × 10^1^	2.84 × 10^−1^	2.21 × 10^1^	1.53 × 10^0^
*m*	2.97 × 10^−2^	1.01 × 10^−3^	3.36 × 10^−2^	1.57 × 10^−3^	3.37 × 10^−2^	1.20 × 10^−3^	1.02 × 10^−2^	1.03 × 10^−3^

*b, COVID-19 transmission rate; m, death probability; r, contact reduction; t_i_, start of intervention policy; Δ, duration of intervention policy; τ_1_, incubation period; τ_2_, period from symptoms to death; τ_3_, period from symptoms to recovery; θ, fraction of notified cases. The model parameters were calibrated using data from the first day with more than 100 cases to 10 days before the pandemic peak for Austria, Germany, and S. Korea. For Italy the model parameters were calibrated from 08/17/2020 to 10 days before the second peak*.

## 3. Results

### 3.1. The Calibrated Model Captures the Peak of the COVID-19 Pandemic

**Figure 2:**
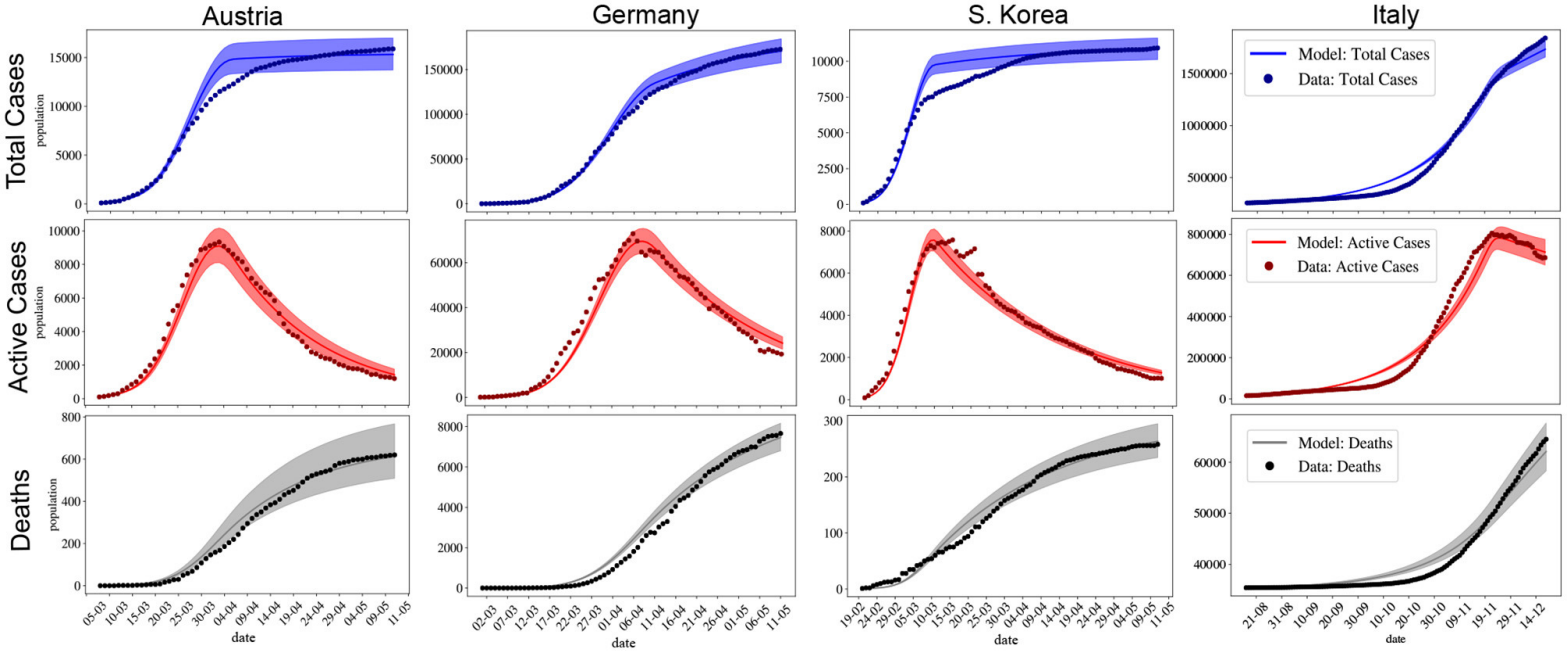
Total number of cases, active cases and deaths for Austria, Germany, South Korea, and Italy. Available data is represented by •. The solid lines indicate the expected value obtained numerically after parameters have been fitted, shaded regions indicate the 95% confidence interval (CI) region. The x-axis is representing days in dd/mm format.

First, to check if the proposed model is able to fit the available data of countries during the peaks. A summary of the inverse UQ analysis results is presented in Table 1, which presents the mean and standard deviation (SD) of the estimated PDFs of the parameters for the three countries. Figure 2 compares the results of the fitted models to the original data for each country. For each time instant *t*, *I*(*t*), *C*(*t*) and *D*(*t*) are PDFs, in response to the process of forward uncertainty quantification. It should be noted that the same model, with different parameters, was able to reproduce the distinct scenarios and peaks of the COVID-19 pandemic in Germany, Austria, Italy, and S. Korea. For the case of Italy, we fitted the recent second peak that has just been reached.

### 3.2. The Models Correctly Forecast the Dynamics of COVID-19 Away From the Peaks

**Figure 3:**
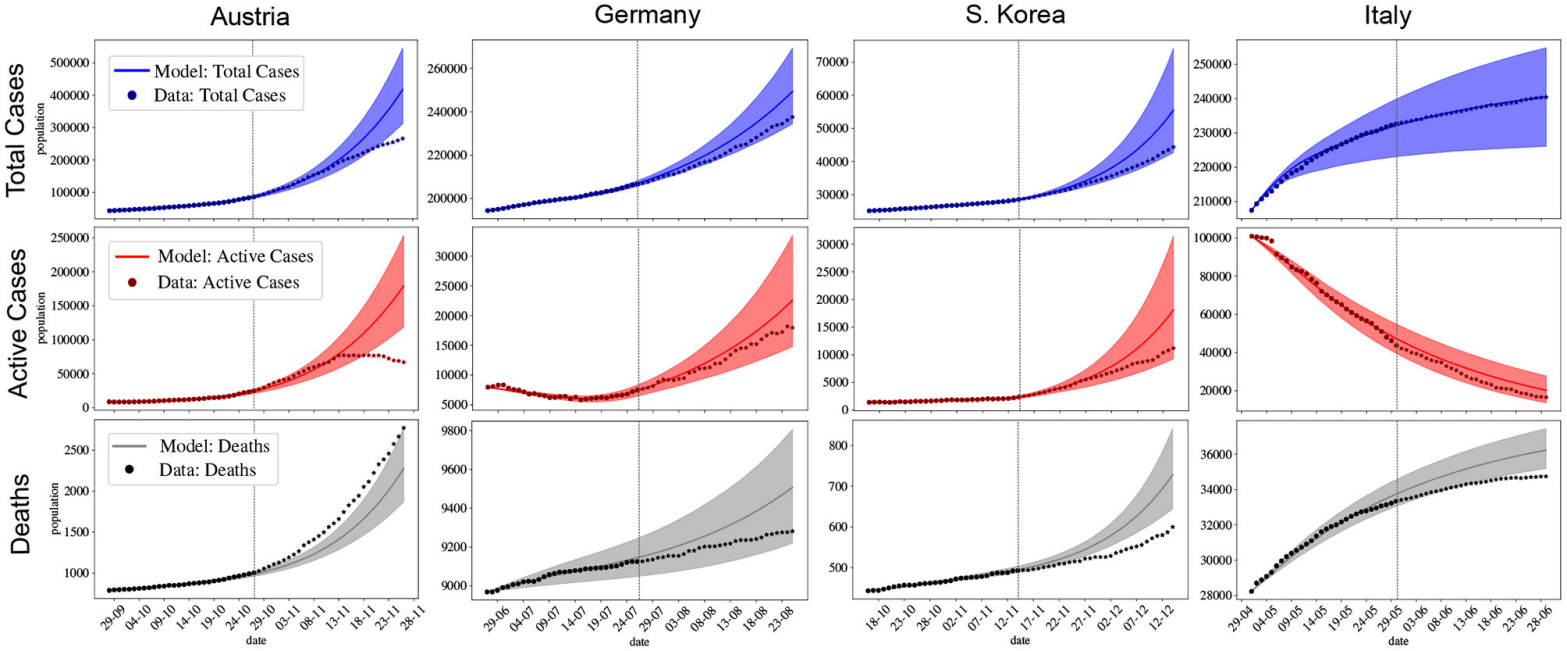
Total number of cases, active cases and deaths for Austria, Germany, South Korea, and Italy. Short forecasts of the dynamics of the pandemic for four countries. Available data is represented by • and ⋆. • represent the days used for fitting (before the vertical dotted line) and ⋆ represent the data that was not considered for fitting (after the vertical dotted line). The solid lines indicate the expected value obtained numerically after parameters have been fitted, shaded regions indicate the 95% confidence interval (CI). The x-axis is representing days in dd/mm format.

Figure 3 shows that the models can correctly forecast the dynamics of COVID-19 for the four countries when away from the peaks. The match between forecasts and real data is observed to last between 20 and 30 days after the fitting phase, which used 30 days for all countries. We have chosen different but all recent phases for these forecasts. The curves' shapes are very distinct, but as long as the forecast is away from the peak, the prediction is within the calculated interval of confidence. Note the particular valley shape captured by the forecast for Germany. Table 2 presents the results of the calibration process.

### 3.3. A Shared Weakness: Forecasting the Peak of COVID-19

As mentioned in the introduction, a literature review shows that many models fail to forecast the peak of the pandemic (26–28), regardless if they are based on SEIR models (26), SIRD (25) extensions of SEIR with more compartments (28), statistical (33, 34), agent-based (35), machine-learning (36), or chaos-based theory models (27).

**Figure 4:**
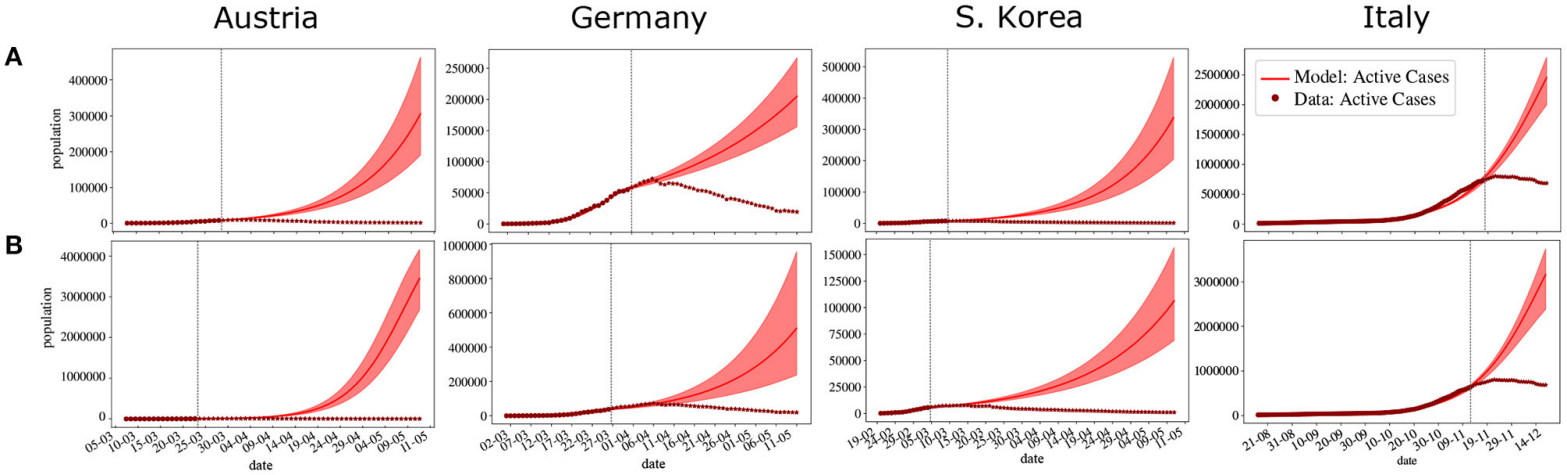
Forecasting the dynamics of the pandemic for four countries based on fitting the model to active cases data available until **(A)** 5 days before the peak **(B)** 10 days before the peak. Available data is represented by • and ⋆. • represent the days used for fitting (before the vertical dotted line) and ⋆ represent the data that was not considered for fitting (after the vertical dotted line). The solid lines indicate the expected value obtained numerically after parameters have been fitted, shaded regions indicate the 95% confidence interval (CI). The x-axis is representing days in dd/mm format.

The same happens with our model. The following experiment was performed: the parameters were adjusted again using data available for active cases until 10 days before achieving the pandemic peak. Then, we tried to predict the number of active cases in the next days. The same experiment was then performed adjusting the data available until 5 days before achieving the pandemic peak. We chose to calibrate the models up to 5 or 10 days before the peak to show that even when it is very close to occurring, forecasts may fail. A summary of the results of the inverse UQ analysis is presented in Tables 3, 4, presenting the mean and standard deviation of the estimated PDFs of the parameters adjusted for the three countries using data until 5 and 10 days, respectively, before the pandemic peak. Figure 4 presents the forecasts. As one can observe, even in the case where the model was adjusted using data available 5 days before the peak, the calibrated model was not able to correctly predict the peaks.

### 3.4. The Main Source of Error: Misestimation of Transmission Rates Near the Peak

In this section we investigate the possible sources of errors when forecasting the peaks of COVID-19. First, by comparing the inverse uncertainty quantification results presented in Tables 1, 3, 4 we can observe that the main difference between the simulations that capture the peaks (Figure 2) and those that do not (Figure 4) lies on the estimation of the parameters that describe the time-varying transmission rate (*a*(*t*)): *t*_*i*_, *r*, and Δ. When the peak is well capture by the model, *a*(*t*) reduces significantly near the peak.

We continue this investigation by performing a sequence of four backward projection experiments, named E1 to E4. To describe these experiments, let *P* denotes the day of the peak in the corresponding country considered in this study. The first experiment (E1) calibrates the model parameters up to 5 days before the peak, as performed before, but focusing near the peak, from *P* − 10 to *P* − 5 days. For the second experiment (E2), we expand the model calibration toward the peak, from *P* − 10 to *P*. We continue expanding the window surrounding the peak in the third experiment (E3), performing the adjustment from *P* − 10 to *P* + 10 days. Finally, in the last experiment (E4), the last calibration is performed from *P* − 10 to *P* + 15 days. Then we compare all the parameters to check those that vary most between the different calibrations, which include the one we used before that failed forecasting the peak (E1, *P* − 10 to *P* − 5) and the one that captures the peak (E4). Once again, the main difference between the different experiments' estimated parameters was on those that describe the time-varying transmission rate (*a*(*t*)): *t*_*i*_, *r*, and Δ.

**Figure 5:**
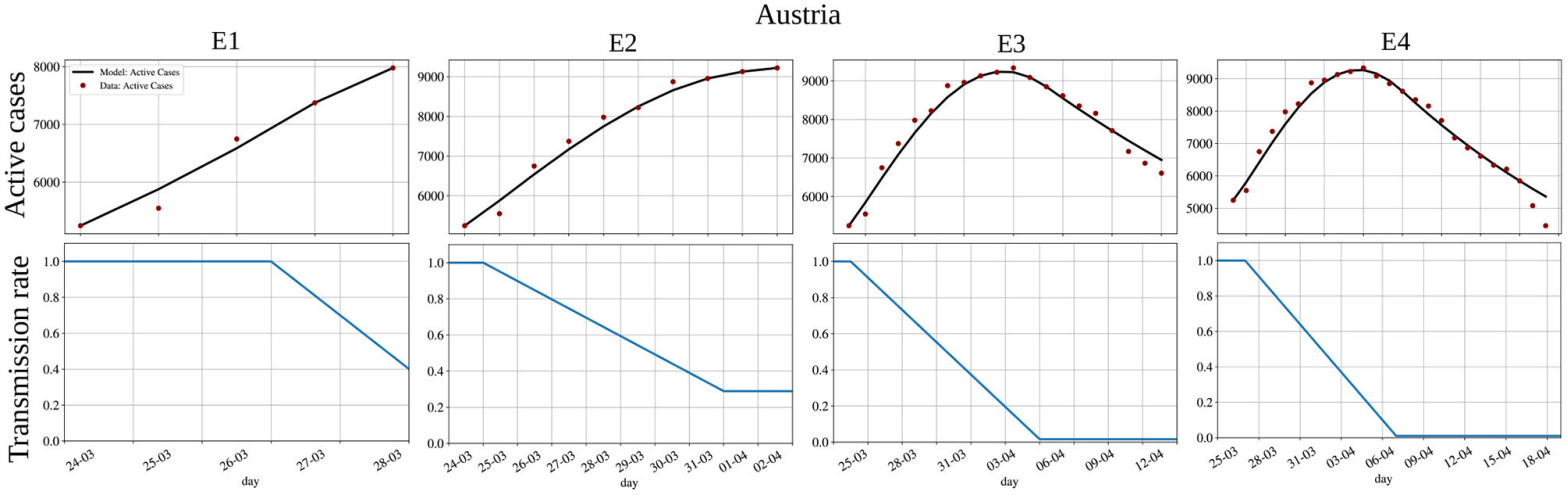
Backward projection of the pandemic's peak for Austria: active cases **(top)** and transmission rate *a*(*t*) **(bottom)**. Fitting the model to active cases data until (E1) 5 days before the peak, (E2) the peak, (E3) 10 days after the peak, and (E4) 15 days after the peak. The x-axis is representing days in dd/mm format.

Figure 5 presents the experiments E1-E4 for Austria and each corresponding estimated *a*(*t*). In this case, we note that from the adjusted and experimental data of E1 (active cases), there is little to no indication that we are slowing down and reaching the peak. Also, by comparing the shapes of the estimated *a*(*t*), we observe that only 10 days after the peak (E3), the crucial information on how the transmission rate evolves before the peak converges [the shapes of *a*(*t*) obtained in E3 and E4 are nearly the same].

**Figure 6:**
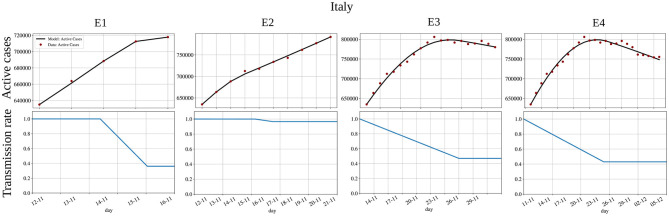
Backward projection of the pandemic's peak for Italy: active cases **(top)** and transmission rate *a*(*t*) **(bottom)**. Fitting the model to active cases data until (E1) 5 days before the peak, (E2) the peak, (E3) 10 days after the peak, and (E4) 15 days after the peak. The x-axis is representing days in dd/mm format.

Figure 6 presents the experiments E1-E4 for Italy and each corresponding estimated *a*(*t*). In this case, we note that even from the adjusted and experimental data of E2 (active cases from *P* − 10 to *P*), there is little to no indication that we are slowing down and reaching the peak. Again, by comparing the shapes of the estimated *a*(*t*), we observe that only 15 days after the peak (E4), we can correctly estimate the shape of *a*(*t*) before the peak.

### 3.5. Projections Considering Different Scenarios of Transmission Rate Reduction

The last experiment makes projections considering different scenarios of transmission rate reduction. Again, the parameters were adjusted using data available for active cases until 5 and 10 days before achieving the peak (in the active cases) for the four countries. The idea here is to evaluate the impact of different shapes of *a*_*p*_(*t*) after the calibration, i.e., different scenarios of transmission rate reduction.

Since the projection is done considering the same dates used for the forecasts, the values in Tables 3, 4 are the same for both experiments. In the projections we consider two distinct values for *r*_*f*_, 0.10 and 0.05, i.e., two different final values for *a*_*p*_(*t*), and two distinct values for Δ_*f*_, 7 and 14. All projections considered *t*_*i*_ = 0. Therefore, were are considering different scenarios where transmission rate reduces after the calibration *a*_*p*_(*t*), decreasing to 0.10 or 0.05, after 1 or 2 weeks.

**Figure 7:**
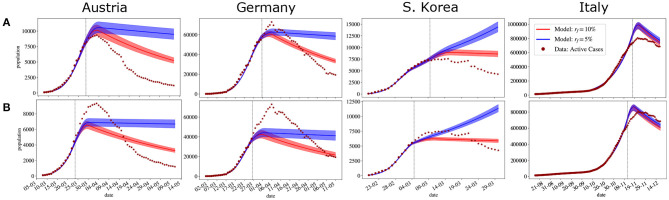
Projections considering different isolation policies for Austria, Germany, S. Korea, and Italy are considering data until 5 days before the peak **(A)** and 10 days before the peak **(B)**. All projections considered Δ_*f*_ = 7 days. Projections in blue and red considered a final contact reduction of 10% and 5%, respectively. • represents the days used for fitting (before the vertical dotted line), and ⋆ represent the days that were not considered for the fitting (after the vertical dotted line). The solid lines indicate the expected value obtained after parameters were fitted; shaded regions indicate the 95% confidence interval (CI). The x-axis represents days in dd/mm format.

**Figure 8:**
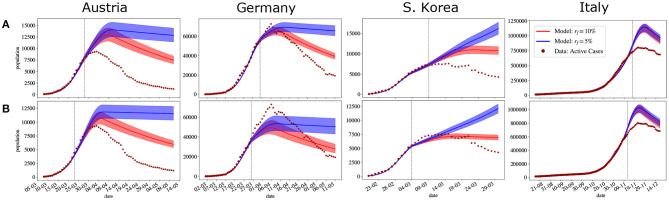
Projections considering different isolation policies for Austria, Germany, S. Korea, and Italy are considering data until 5 days before the peak **(A)** and 10 days before the peak **(B)**. All projections considered Δ_*f*_ = 14 days. Projections in blue and red considered a final contact reduction of 10% and 5%, respectively. • represents the days used for fitting (before the vertical dotted line), and ⋆ represent the days that were not considered for the fitting (after the vertical dotted line). The solid lines indicate the expected value obtained after parameters were fitted; shaded regions indicate the 95% confidence interval (CI). The x-axis represents days in dd/mm format.

Figure 8 presents the projections for the four countries with Δ_*f*_ = 7. The peaks becomes visible when projecting a more significant reduction in the transmission rates, i.e., with *r* = 0.05. Figure 7 presents the projections with Δ_*f*_ = 14. The results are similar to the previous projections. The main difference is that the peaks are higher and occur further ahead. These results support that the fastest way to control the pandemic is with strict mitigation policies that can significantly reduce the transmission rate in a short period.

## 4. Discussion

First of all, one should observe that the model used in this work can reproduce the dynamics of COVID-19 for distinct countries. The pandemic peak for all countries considered here was utterly determined since the adjusted model captures both the day in which the peak occurs as well as its maximum value, as shown in Figure 2. Figure 3 also shows that the model can be very useful in forecasting the dynamics of COVID-19.

There is, however, one main weakness of this model: forecasts near the pandemic peak usually fail. In Figure 4, the same method was applied to adjust the parameters of the model, but this time we did not use the entire dataset. The model was adjusted using data until 5 and 10 days before the peak of the pandemic. As can be observed in Figure 4, the forecasts overestimate the number of active cases and mispredict the peaks by more than a month.

In this work, we have used many sophisticated tools base on forward and inverse UQ to identify the source of this problem. First, we compared the inverse UQ results presented in Tables 1, 3, 4 and observed that the main difference between the simulations that capture the peaks (Figure 2) and those that do not (Figure 4) lies on the estimation of the parameters that describe the time-varying transmission rate (*a*(*t*)): *t*_*i*_, *r*, and Δ. When the peak is well-captured by the model, *a*(*t*) reduces significantly near the peak. This reduction in the transmission rate is key in forecasting the pandemic peak. Long forecasts of the pandemic peak are therefore undermined by the lack of models that can forecast changes in the transmission rate, i.e., how a particular society behaves, changes of mitigation policies, or how society chooses to respond to them.

In addition, we performed a sequence of four backward projection experiments, named (E1) to (E4). The first experiment (E1) calibrates the model parameters up to 5 days before the peak. The second one (E2) calibrates it up to the peak, (E3) up to the peak plus 10 days, and (E4) up to the peak plus 15 days. Then we compared all the parameters to check those that vary most between the different calibrations. Once again, the main difference between the different experiments' estimated parameters was on those that describe the time-varying transmission rate, *a*(*t*). Figures 5, 6 show also that the shape of the estimated *a*(*t*) only converges to the correct one when using epidemic data that includes many days after the peak. These results clearly show a delay between changes in the transmission rate and their impact on the curve of active cases, which is about 15 days. This is intrinsic to the phenomenon and how it affects epidemic data: a new case is usually only reported after an incubation period followed by a delay associated with the test.

The presence of this delay suggests that the challenging task of forecasting the pandemic peak might require additional data and constant monitoring to capture the transmission rate better.

The aforementioned results clearly show how inverse UQ and backward projections can provide important information on the dynamics of the COVID-19 pandemic. Finally, we have also performed forward projections to assess different scenarios of transmission rate reduction. Figures 7, 8 show how significant changes in the transmission rate impacts the dynamics and influences the location of the peaks. In addition, these results support that the fastest way to control the pandemic is with strict mitigation policies that can significantly reduce the transmission rate in a short period.

In summary, our results highlight how mathematical models can help the fight against the COVID-19 pandemic: by characterizing important parameters that dictate the dynamics of the pandemic, as performed before in our previous work (20); and via projections, when different scenarios can be tested and used for decision-making. In addition, they suggest that forecasting the peaks of COVID-19 can be quixotic due to the challenges that involve a precise estimation of how the transmission rate evolves.

## Data Availability Statement

The dataset used for this study can be found in the CSSEGIS and Data at: http://github.com/CSSEGISandData/COVID-19.

## Author Contributions

RF, BM, and RS: software, methodology, and formal analysis. BdM and JM: formal analysis. JdO: software and formal analysis. ML: methodology and formal analysis. RW: conceptualization, software, methodology, and formal analysis. All authors: writing the original draft, review and editing, and have read and approved the final manuscript.

## Conflict of Interest

The authors declare that the research was conducted in the absence of any commercial or financial relationships that could be construed as a potential conflict of interest.
